# The Slo1 Y450F Substitution Modifies Basal Function and Cholesterol Response of Middle Cerebral Artery Smooth Muscle BK Channels in a Sexually Dimorphic Manner

**DOI:** 10.3390/ijms26083814

**Published:** 2025-04-17

**Authors:** Elizabeth H. Schneider, Alex M. Dopico, Anna N. Bukiya

**Affiliations:** Department of Pharmacology, Addiction Science, and Toxicology, College of Medicine, University of Tennessee Health Science Center, Memphis, TN 38103, USA

**Keywords:** patch clamp, cerebral artery myocytes, vessel diameter, methyl-β-cyclodextrin (MβCD), MaxiK channel, *KCNMA1*

## Abstract

Calcium- and voltage-gated potassium channels of large conductance (BK channels) in smooth muscle (SM) act as part of a negative feedback mechanism on SM contraction and associated decrease in cerebral artery diameter. Functional BK channels result from tetrameric association of α subunits encoded by *KCNMA1* (*Slo1*). Ionic current from slo1 channels is inhibited by cholesterol in artificial lipid bilayers, an effect significantly reduced by the slo1 Y450F substitution. Whether such substitution affects cholesterol action on cerebral artery SM BK channel function and diameter remains unknown. Using the *KCNMA1Y450F* knock-in (K/I) mouse, we determined the effect of cholesterol enrichment on BK currents in native SM cells from middle cerebral artery using patch-clamp electrophysiology and the artery diameter ex vivo response to cholesterol. Results show that the *KCNMA1Y450F* K/I mutation modifies both basal function and the channel’s response to cholesterol enrichment. Such modifications are detectable solely in SM cells from males, demonstrating sexual dimorphism. Unexpectedly, the modifications introduced by the Y450F substitution do not translate into observable changes in middle cerebral artery diameter ex vivo, suggesting that mechanisms at the SM level compensate for changes driven by the *KCNMA1* point mutation under study.

## 1. Introduction

Cerebral arteries provide the brain with essential oxygen and nutrients. Large cerebral arteries branching from the Circle of Willis—basilar and anterior, middle (MCA), and posterior cerebral arteries—perfuse both cortical and subcortical structures, the latter through parenchyma-penetrating vessels [1,2]. The wall of large and resistance-sized cerebral arteries is composed of supporting external adventitia; tunica media, largely made of smooth muscle (SM) cells; and the tunica intima, composed of a single layer of endothelial cells [3]. It is the SM layer which is responsible for the generation of myogenic tone for the maintenance of consistent blood perfusion to the brain within a wide range of systemic blood pressure values [4]. In the cerebrovascular SM cells, calcium- and voltage-gated potassium channels of large conductance (BK type) act as a negative feedback mechanism on depolarization and increased intracellular calcium-induced SM contraction, leading to SM relaxation and vasodilation [5,6,7].

In mammals, BK channels are encoded by the *KCNMA1* or *Slo1* gene. Protein products of *KCNMA1* are ubiquitously expressed in mammalian tissue [8,9]. Slo1 proteins, also known as BK channel α subunits, tetramerize to form transmembrane functional channels, referred to as slo1 channels. However, native BK channels in most tissues include a variety of auxiliary subunits of β and γ types, which show highly specific tissue expression. These subunits form complexes with slo1 channels and thus modify the channel phenotype. Given the tissue-specific expression of these regulatory subunits, the resulting pleiotropic phenotype of native BK channels allows these channels to suit cell physiology in a tissue-specific manner. Thus, the high expression of β_1_ subunits in vascular SM allows BK channels to sense increases in calcium during SM contraction and exert their negative feedback role on SM contraction and vasoconstriction [9,10,11].

In addition, the surrounding lipid environment of the membrane where the BK channel is embedded adds to the diversity of BK channel phenotypes and serves as a major regulator of BK channel function. Cholesterol is an essential component of animal membranes [12,13,14,15,16] and has been demonstrated to be a ligand of a variety of proteins, including ion channels [17,18,19,20,21]. The plasma membrane concentration of cholesterol varies between 20 and 50 mol% based on a variety of factors, including diet, cell type, and artery type [22]. In artificial lipid bilayers, where the cholesterol molar fraction can be tightly controlled, it has been shown that cholesterol at concentrations that match those in native plasma membranes (33 mol%) decreases channel open probability (Po) and unitary current amplitude of homotetrameric slo1 channels [23,24]. Reduction in Po by cholesterol is ablated by the Y450F mutation, suggesting that Y450 participates in cholesterol modulation of slo1 channel activity [24]. The role of Y450 in cholesterol sensing by heteromeric BK channels in the complex proteolipid environment of native membranes, however, remains unknown. On the other hand, in compensation to the inhibitory action of cholesterol on slo1 channels in planar lipid bilayers, cholesterol enrichment of MCA SM cells has been reported to increase BK channel activity in male rats [25]. This activation, which overrules the direct inhibition of cholesterol on slo1 channels, is due to cholesterol-driven increase in the plasmalemma fraction of β1 subunits, where they form complexes with BK α tetramers [25]. Based on the antagonistic effects of cholesterol actions on slo1 channels in cell-free environments vs. native BK channels in intact SM cells, if cholesterol inhibition of slo1 channels persists in their native MCA SM counterparts, we hypothesize that cholesterol enrichment of SM cells from *KCNMA1Y450F* animals would potentiate BK currents to a degree higher than that reported in native channels from WT mice. Thus, in the present study, using the *KCNMA1Y450F* mouse, we set out to determine the influence of this mutation on the modulation of native middle cerebral artery SM BK channels by cholesterol and the possible influence of such mutation on the response of middle cerebral artery diameter to cholesterol enrichment.

## 2. Results

### 2.1. Cholesterol Enrichment of Middle Cerebral Arteries (MCA) Using Methyl-β-Cyclodextrin (MβCD) as a Vehicle

**Figure 1:**
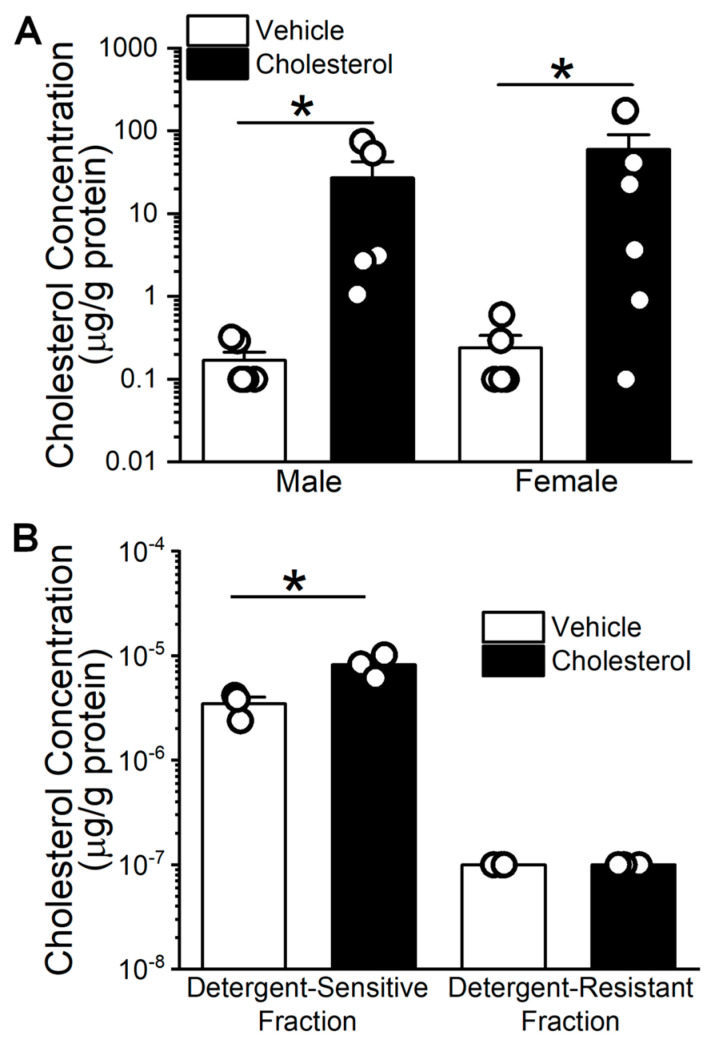
Cholesterol levels in middle cerebral arteries (MCA) of wild-type (WT) *KCNMA1* mice (C57BL/6J). (**A**) Cholesterol enrichment increases overall cholesterol levels in male and female mouse MCAs. Prior to cholesterol determination, MCAs were dissected from mouse brain and incubated in phosphate-buffered saline containing either 5 mM MβCD (Vehicle) or 6.25 mM cholesterol/5 mM MβCD (cholesterol, cholesterol-enriching solution) for 15 min. Then, MCA tissue was lysed, and cholesterol concentration was measured and normalized to protein amount. Compared to vehicle, MCAs from both male and female mice were significantly enriched with cholesterol upon incubation in cholesterol-enriching solution. * *p* = 0.016 for both sexes in nonparametric Mann–Whitney U test. (**B**) Cholesterol enrichment increases cholesterol level in detergent-sensitive, but not detergent-resistant, membrane fraction of mouse cerebral arteries. Data were obtained upon a 15 min incubation of male WT *KCNMA1* mouse (C57BL/6J) cerebral arteries in cholesterol-enriching solution. For details of fraction separation, please refer to Material and Methods. * *p* = 0.039 in nonparametric Mann–Whitney U test.

Cyclodextrins are commonly used to manipulate cholesterol levels in cellular membranes [26]. After dissecting the MCA as described in the Methods, we determined the ability of male and female mouse MCA to be enriched in cholesterol by these agents. Thus, MCA cholesterol levels were quantified following a 15 min incubation in 6.25 mM cholesterol using 5 mM MβCD as a vehicle. In parallel, MCA was incubated in cholesterol-free MβCD (5 mM) as a vehicle-containing control. Figure 1A shows that MCAs from both male and female are significantly enriched with cholesterol based on a Mann–Whitney U nonparametric test (*p* = 0.016 in both sexes). Cholesterol enrichment of MCA from males averages 27.05 ± 34.71 μg/g of protein vs. 0.11 ± 0.15 μg/g of protein in controls. Similarly, cholesterol enrichment of MCA from females averages 59.72 ± 80.00 μg/g of protein vs. over 0.18 ± 0.26 μg/g of protein in controls. These results demonstrate the efficiency of our method in enriching MCA with cholesterol and the fact that this efficiency is independent of animal sex.

Notably, the increase in total cholesterol level upon a 15 min incubation of mouse cerebral arteries in cholesterol-enriching solution is largely driven by a significant increase in cholesterol amount within the detergent-sensitive fraction (3.47 × 10^−6^ ± 9.46 × 10^−7^ to 8.20 × 10^−6^ ± 2.03 × 10^−6^ μg/mg protein), but not the detergent-resistant fraction (Figure 1B).

### 2.2. Effect of Cholesterol Enrichment on Function of Middle Cerebral Artery (MCA) Smooth Muscle (SM) Native BK Channels and Role of the slo1Y450 Substitution

**Figure 2:**
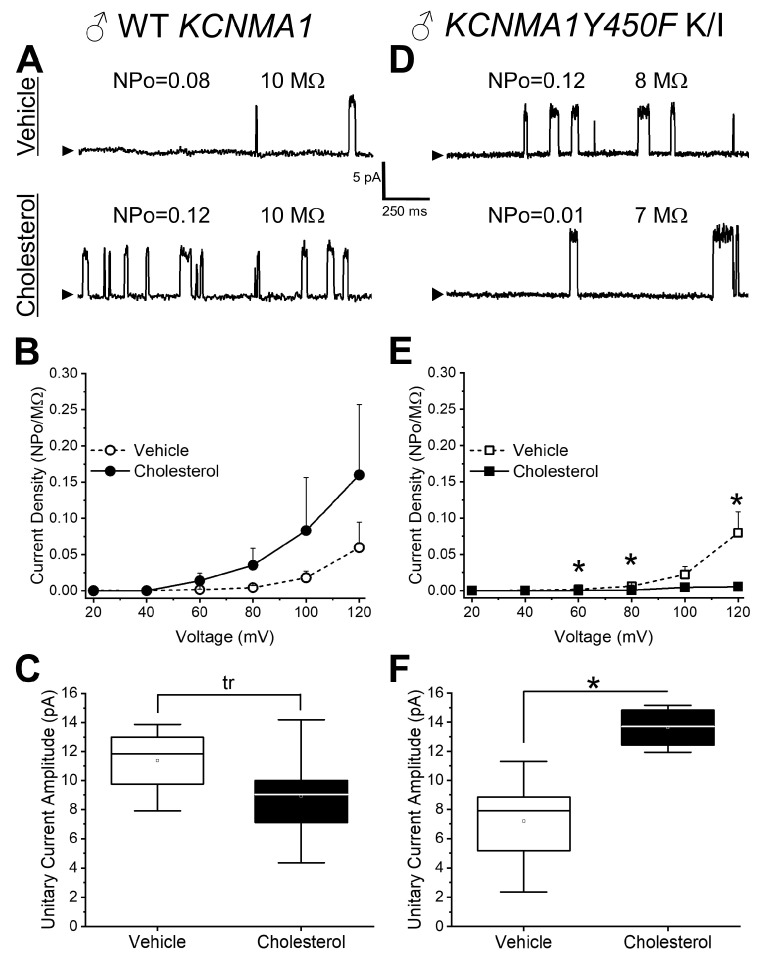
Cholesterol enrichment has mild effect on BK currents in myocytes from male WT *KCNMA1* middle cerebral arteries, yet decreases current density and increases unitary current amplitude of male *KCNMA1Y450F* K/I. (**A**) Representative traces of cell-attached patch-clamp recordings at +80 mV from myocytes that were freshly isolated from middle cerebral arteries (MCAs) of male WT *KCNMA1* mice. Top recording is obtained following myocyte incubation in vehicle-containing bath solution, while bottom trace depicts currents following incubation in cholesterol-enriching solution. Here and in (**D**), NPo represents BK channel activity where N reflects number of channel opening levels, while Po describes probability of a single channel being open. MΩ: pipette tip resistance. (**B**) BK current density (normalized over pipette resistance)–voltage plots obtained from myocytes incubated in vehicle and cholesterol-enriching solutions. Myocytes were obtained from MCAs of male WT *KCNMA1*. Normalized current density reflects channel activity (NPo) divided by pipette size (MΩ). The latter was determined based on the current amplitude passing via pipette placed in bath solution and in response to a 10 mV pulse. Pipettes with resistance below 5 MΩ or exceeding 10 MΩ were not used for data acquisition. (**C**) Unitary BK current amplitude at +80 mV from myocytes incubated in vehicle as opposed to cholesterol-enriching solution. Myocytes were obtained from MCAs of male WT *KCNMA1* mice. A high R-squared value (R^2^ = 0.86) and low but not significant *p*-value (*p* = 0.072) suggest a statistical trend (tr). (**D**) Representative traces of cell-attached patch-clamp recordings at +80 mV from myocytes that were freshly isolated from MCAs of male *KCNMA1Y450F* K/I mice. Top recording is obtained following myocyte incubation in vehicle-containing bath solution, while bottom trace depicts currents following incubation in cholesterol-enriching solution. (**E**) BK current density–voltage plots obtained from myocytes incubated in vehicle and cholesterol-enriching solutions. Myocytes were obtained from MCAs of male *KCNMA1Y450F* K/I. * *p* = 0.004, 0.005, and 0.007, respectively, with increasing voltage. Statistical analysis was performed using nonparametric Mann–Whitney U test. (**F**) Unitary BK current amplitude at +80 mV from myocytes incubated in vehicle as opposed to cholesterol-enriching solution. Myocytes were obtained from MCAs of male *KCNMA1Y450F* K/I mice. * *p* = 0.048 by nonparametric Mann–Whitney U test.

To determine at a single-channel resolution the effect of cholesterol on native BK channels in intact MCA SM cells isolated from a male mouse, we conducted patch-clamp electrophysiology using the cell-attached configuration. Myocytes were isolated from MCAs and incubated for 15 min in either 5 mM MβCD (vehicle) or 6.25 mM cholesterol/5 mM MβCD (cholesterol-enriching solution) prior to patching. After a 10 min washout of the cell upon MβCD or cholesterol-enriching solution treatment, BK currents were recorded at positive transmembrane voltages to ensure measurable levels of BK channel steady-state activity (NPo). The identities of the currents were validated by their sensitivity to the BK channel blocker paxilline (1 μM) [27] (Supplementary Figure S1). As membrane size changes from patch to patch and cholesterol introduction into a membrane modifies its specific capacitance (i.e., independently of membrane area), we decided to use NPo/pipette tip resistance as a measure of current density (dependent variable). In native MCA SM channels from WT males, there is no significant difference in current density between the vehicle-containing control and cholesterol enrichment treated cells, albeit the data show an actual trend towards potentiation by cholesterol (Figure 2A,B). In turn, cholesterol enrichment evokes a mild decrease in unitary current amplitude, which shows a statistical trend. The decrease in current amplitude is consistent with previous reports (see Section 3). In contrast to these findings, a significant decrease in current density is observed in cholesterol-enriched MCA SM cells from the *KCNMA1Y450F* mouse when evaluated at +60, +80, and +120 mV (Figure 2E). Remarkably, under control conditions, these mice show a basal unitary current that is smaller (7.19 ± 2.86 pA) than that of their WT counterparts (11.37 ± 2.51 pA) (*p* = 0.048 in Mann–Whitney U nonparametric test; Figure 2C,F). Thus, this decrease seems to mask any further decrease in unitary current amplitude by cholesterol, leading to an apparent increase in the unitary conductance of BK channels from *KCNMA1Y450F* mice in response to cholesterol enrichment (Figure 2F). For instance, at +80 mV, unitary current amplitude increases from 7.19 ± 2.86 pA (vehicle control) to 13.63 ± 1.48 pA (cholesterol enrichment) (*p* = 0.048 in Mann–Whitney U nonparametric test).

**Figure 3:**
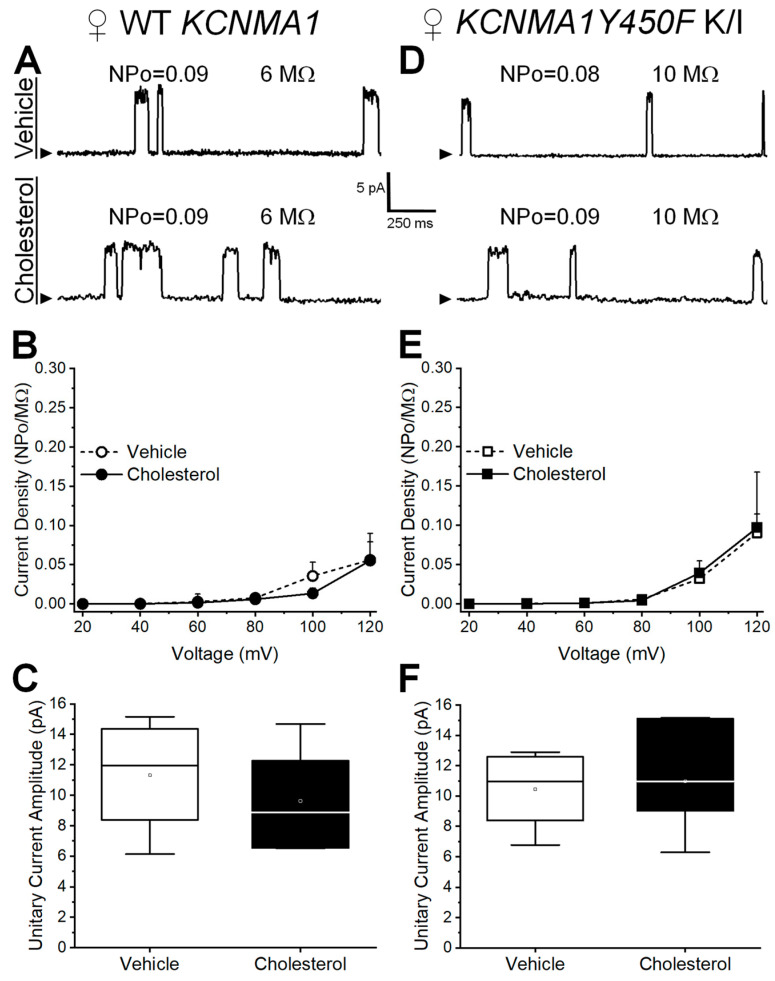
Cholesterol enrichment has no effect on BK current density or unitary current amplitude in myocytes isolated from middle cerebral arteries (MCA) of female WT and *KCNMA1Y450F* K/I mice. (**A**) Representative traces of cell-attached patch-clamp recordings at +80 mV from myocytes that were freshly isolated from MCAs of female WT *KCNMA1* mice. Top recording is obtained following myocyte incubation in vehicle-containing bath solution, while bottom trace depicts currents following incubation in cholesterol-enriching solution. Here and in (**D**), NPo represents BK channel activity, where N reflects number of channel opening levels and Po describes probability of a single channel being open. MΩ: pipette tip resistance. (**B**) BK current density–voltage plots obtained from myocytes incubated in vehicle and cholesterol-enriching solutions. Myocytes were obtained from MCAs of female WT *KCNMA1*. Current density was obtained by normalizing channel activity NPo over pipette resistance. (**C**) Unitary BK current amplitude at +80 mV from myocytes incubated in vehicle as opposed to cholesterol-enriching solution. Myocytes were obtained from MCAs of female WT *KCNMA1* mice. (**D**) Representative traces of cell-attached patch-clamp recordings at +80 mV from myocytes that were freshly isolated from MCAs of female *KCNMA1Y450F* K/I mice. Top recording is obtained following myocyte incubation in vehicle-containing bath solution, while bottom trace depicts currents following incubation in cholesterol-enriching solution. (**E**) BK current density–voltage plots obtained from myocytes incubated in vehicle and cholesterol-enriching solutions. Myocytes were obtained from MCAs of female *KCNMA1Y450F* K/I. (**F**) Unitary BK current amplitude at +80 mV from myocytes incubated in vehicle as opposed to cholesterol-enriching solution. Myocytes were obtained from MCAs of female *KCNMA1Y450F* K/I mice.

Cell-attached patch-clamp electrophysiology was also performed on MCA SM cells from female mice. In MCA myocytes from WT *KCNMA1* female mice, current density is unchanged by cholesterol enrichment when compared to the vehicle-containing control at a wide range of transmembrane voltages (Figure 3A,B). Thus, the trend for cholesterol to introduce a leftward shift to the current density–voltage plot found in WT males (Figure 2B) is lost in WT females. Remarkably, data from MCA myocytes of *KCNMA1Y450F* K/I female mice further differ from those of male *KCNMA1Y450F* K/I. First, unlike males (Figure 2E), current density in females remains insensitive to cholesterol enrichment at the entire range of transmembrane voltages tested (Figure 3E). Moreover, the decrease in unitary current amplitude introduced by the Y450F substitution as observed in males (Figure 2C vs. Figure 2F, empty boxes) is attenuated in females (Figure 3C vs. Figure 3F, empty boxes). Thus, unitary current amplitude in female *KCNMA1Y450F* K/I animals appears insensitive to cholesterol enrichment (Figure 3F). In summary, the *KCNMA1Y450F* mutation modifies both the basal function and cholesterol response of native BK channels when studied in intact SM cells isolated from MCAs, and these modifications are sexually dimorphic.

### 2.3. Cholesterol Enrichment Effect on Middle Cerebral Artery Diameter and Influence of the KCNMA1Y450F Substitution

To address the effect of cholesterol on MCA SM function, we determined the effect of cholesterol on the diameter of mouse MCA segments pressurized ex vivo. As SM constitutes the largest mass of cerebral artery tissue [3], removing the endothelium allows for the study of biological processes that are largely determined by the vascular SM itself [28]. Thus, MCAs from male mice were de-endothelialized and pressurized to 60 mmHg to trigger the development of myogenic tone. After a stable tone was established, MCAs were extralumenally perfused with either 5 mM MβCD (vehicle) or 6.25 mM cholesterol/5 mM MβCD (cholesterol-enriching solution). Upon their washout, the health of each artery was verified with perfusion of 60 mM KCl, which is commonly used to evaluate depolarizing-induced constriction of arterial SM. Only arteries that constricted 10% or more to this treatment were used for data analysis. There are no statistically significant differences in the degree of KCl-evoked constriction by WT vs. *KCNMA1Y450F* K/I MCA whether evaluated in males or females (Supplementary Figure S2). This seems to indicate that sex and the *KCNMA1Y450F* mutation are not major determinants of de-endothelialized MCA ex vivo constriction by depolarization.

**Figure 4:**
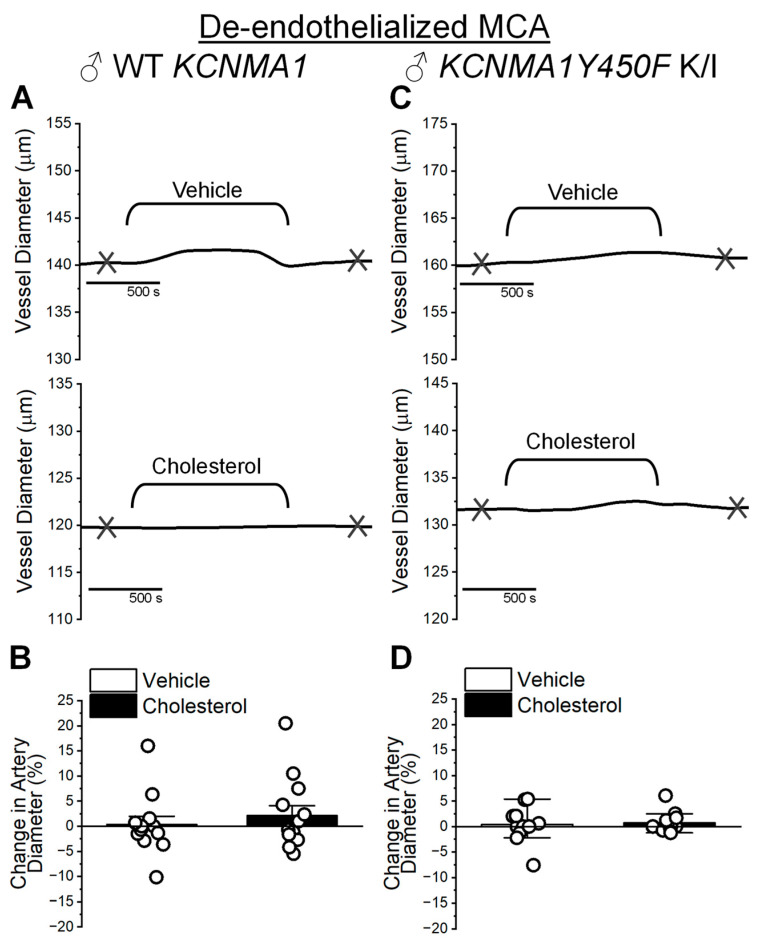
Cholesterol enrichment fails to alter diameter of de-endothelialized middle cerebral arteries (MCAs) from male mice of WT *KCNMA1* and *KCNMA1Y450F* K/I genotypes. (**A**) Representative traces of MCA diameter over perfusion with 5 mM MβCD (vehicle, top trace) or 6.25 mM cholesterol/5 mM MβCD (cholesterol, bottom trace) solutions. MCA was dissected from male WT *KCNMA1* mouse, subjected to endothelium removal, and pressurized at 60 mmHg. Here, and in (**C**), crisscross marks depict time points at which diameter measurements were obtained for plotting. Measurement of base diameter was obtained prior to perfusing pressurized artery with vehicle or cholesterol-enriching solution. Measurements of the effect of cholesterol-enriching perfusion and time-matched vehicle control were obtained 10 min following washout of respective solutions from the pressurized artery chamber. (**B**) Bar graph shows average and individual artery responses to vehicle or cholesterol-enriching solutions. MCAs were obtained from male WT *KCNMA1* mice. Here and in (**D**), change in artery diameter shows change in diameter triggered by vehicle perfusion or cholesterol enrichment as percent of base diameter before perfusion with vehicle or cholesterol-enriching solution. (**C**) Representative traces of MCA diameter over perfusion with 5 mM MβCD (vehicle, top trace) or 6.25 mM cholesterol/5 mM MβCD (cholesterol, bottom trace) solutions. MCA was dissected from male *KCNMA1Y450F* K/I mouse and pressurized at 60 mmHg. (**D**) Bar graph shows average and individual artery responses to vehicle or cholesterol-enriching solutions. MCAs were obtained from male *KCNMA1Y450F* K/I mice.

Consistent with the fact that cholesterol enrichment does not exert a statistically significant effect on BK currents in myocytes from male WT MCAs, artery diameter does not change upon the application of cholesterol-enriching solution (Figure 4A,B). Following the timeline of data acquisition from patch-clamp experiments on isolated myocytes (Figure 2A–C), diameter measurements were collected 10 min after vehicle or cholesterol-enriching solution was washed out from the artery chamber. Change in MCA diameter increases by 0.68 ± 1.65%, whereas MCA diameter change following cholesterol enrichment averages 4.2 ± 2.00% compared to the diameter before cholesterol-enriching solution is introduced into the artery chamber (*p* = 0.801 in Mann–Whitney U nonparametric test) (Figure 4B). Despite the effects of cholesterol enrichment on BK current density (trend to increase) and unitary current amplitude (decrease) in male WT myocytes (Figure 2B,C), MCA in *KCNMA1Y450F* myocytes exhibited ablated sensitivity to cholesterol enrichment (Figure 4C,D). In addition, despite the relative increase in unitary conductance observed in male *KCNMA1Y450F* SM BK channels (Figure 2F), diameter changes in MCAs from *KCNMA1Y450F* K/I males average 1.3 ± 0.58% (cholesterol enrichment) and 0.66 ± 1.08% (vehicle control); there is no statistically significant difference between these two groups (Figure 4D; *p* = 0.976 in Mann–Whitney U nonparametric test).

**Figure 5:**
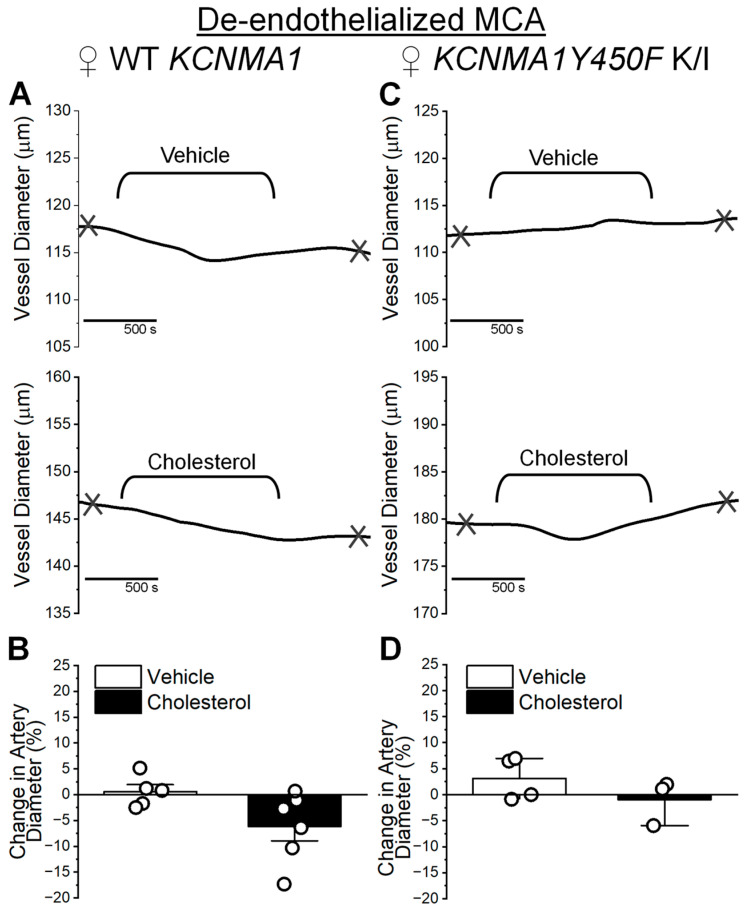
Cholesterol enrichment has no effect on diameter of de-endothelialized MCAs from WT *KCNMA1* and *KCNMA1Y450F* K/I female mice. (**A**) Representative traces of MCA diameter over perfusion with 5 mM MβCD (vehicle, top trace) or 6.25 mM cholesterol/5 mM MβCD (cholesterol, bottom trace) solutions. MCA was dissected from female WT *KCNMA1* mouse, subjected to endothelium removal, and pressurized at 60 mmHg. Here, and in (**C**), crisscross marks depict time-points at which diameter measurements were obtained for plotting. Measurement of base diameter was obtained prior to perfusing pressurized artery with vehicle or cholesterol-enriching solution. Measurements of the effect of cholesterol-enriching perfusion and time-matched vehicle control were obtained 10 min following washout of respective solutions from the pressurized artery chamber. (**B**) Bar graph shows average and individual artery responses to vehicle or cholesterol-enriching solutions. MCAs were obtained from female WT *KCNMA1* mice. Here and in (**D**), change in artery diameter shows change in diameter triggered by vehicle perfusion or cholesterol enrichment as percent of base diameter before perfusion with vehicle or cholesterol-enriching solution. (**C**) Representative traces of MCA diameter over perfusion with 5 mM MβCD (vehicle, top trace) or 6.25 mM cholesterol/5 mM MβCD (cholesterol, bottom trace) solutions. MCA was dissected out of female *KCNMA1Y450F* K/I mouse and pressurized at 60 mmHg. (**D**) Bar graph shows average and individual artery responses to vehicle or cholesterol-enriching solutions. MCAs were obtained from female *KCNMA1Y450F* K/I mice.

From the lack of cholesterol effect on BK channel electrophysiological characteristics in female MCA SM cells (Figure 3), it was expected that there would be no significant effect of cholesterol on the diameter of de-endothelialized arteries. Indeed, MCA from female WT are insensitive to cholesterol-enriching treatment (Figure 5A,B). Diameter changes in MCAs from WT females average −5.72 ± 2.75% (cholesterol enrichment) and 0.45 ± 1.34% (vehicle control) (Figure 5B; *p* = 0.052 in Mann–Whitney U nonparametric test). Accordingly, the *KCNMA1Y450F* mutation does not introduce any change to the female MCA diameter’s unresponsiveness to cholesterol enrichment (Figure 5C,D). Diameter changes in MCAs from *KCNMA1Y450F* K/I females average −0.44 ± 2.51% (cholesterol enrichment) and 1.93 ± 2.07% (vehicle control) (Figure 5D; *p* = 0.343 in Mann–Whitney U nonparametric test). Thus, Figure 5 shows no significant difference in artery diameter, regardless of cholesterol treatment or genotype in MCAs from females.

### 2.4. Cholesterol Enrichment Effect on Cerebral Artery Segments with Intact Endothelium and Role of the KCNMA1Y450F Mutation

**Figure 6:**
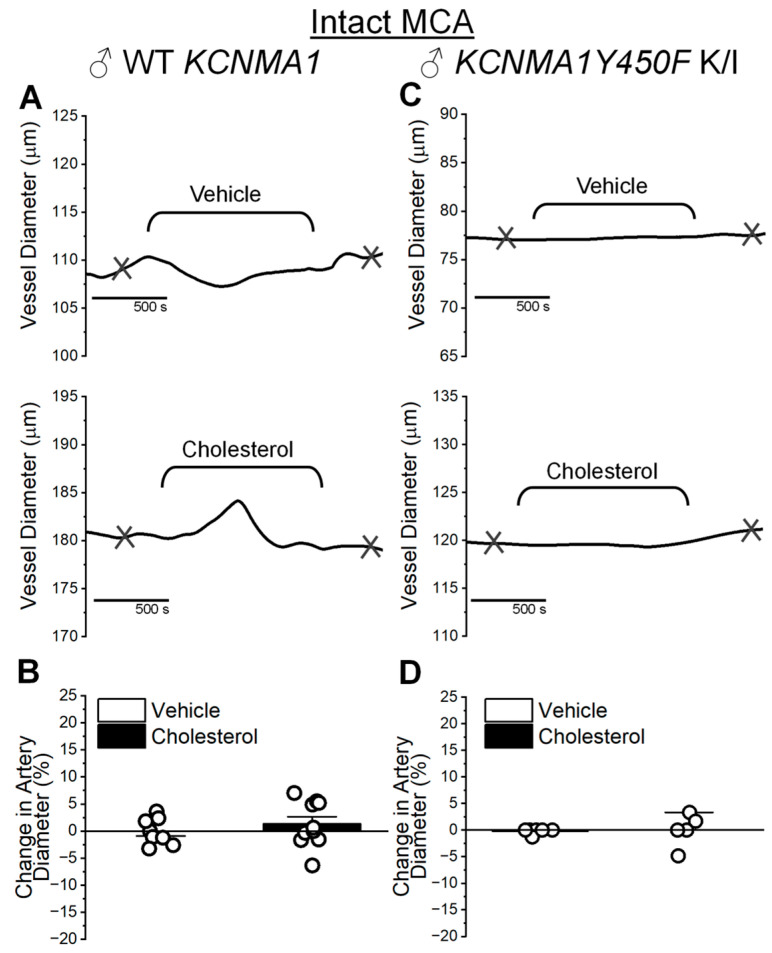
Cholesterol enrichment fails to alter diameter of middle cerebral arteries (MCAs) from male mice of WT *KCNMA1* and *KCNMA1Y450F* K/I genotypes. (**A**) Representative traces of MCA diameter over perfusion with 5 mM MβCD (vehicle, top trace) or 6.25 mM cholesterol/5 mM MβCD (cholesterol, bottom trace) solutions. MCA was dissected out of male WT *KCNMA1* mouse and pressurized at 60 mmHg. Integrity of the endothelium was preserved. Here, and in (**C**), crisscross marks depict time points at which diameter measurements were obtained for plotting. Measurement of base diameter was obtained prior to perfusing pressurized artery with vehicle or cholesterol-enriching solution. Measurements of the effect of cholesterol-enriching perfusion and time-matched vehicle control were obtained 10 min following washout of respective solutions from the pressurized artery chamber. (**B**) Bar graph shows average and individual artery responses to vehicle or cholesterol-enriching solutions. MCAs were obtained from male WT *KCNMA1* mice. Here and in (**D**), change in artery diameter shows change in diameter triggered by vehicle perfusion or cholesterol enrichment as percent of base diameter before perfusion with vehicle or cholesterol-enriching solution. (**C**) Representative traces of MCA diameter over perfusion with 5 mM MβCD (vehicle, top trace) or 6.25 mM cholesterol/5 mM MβCD (cholesterol, bottom trace) solutions. MCA was dissected out of male *KCNMA1Y450F* K/I mouse and pressurized at 60 mmHg. (**D**) Bar graph shows average and individual artery responses to vehicle or cholesterol-enriching solutions. MCAs were obtained from male *KCNMA1Y450F* K/I mice.

To determine whether the endothelium introduces cholesterol sensitivity to the MCA diameter, the next experiments were performed on MCAs pressurized ex vivo with their intact endothelium. Preservation of the endothelium does not confer cholesterol sensitivity to the MCA: diameter changes in MCAs from WT males average 1.34 ± 1.33% (cholesterol enrichment) vs. −0.04 ± 0.87% (vehicle control) (Figure 6A,B; *p* = 0.408 in Mann–Whitney U nonparametric test). Diameter changes in MCAs from *KCNMA1Y450F* males average −0.03 ± 1.37% (cholesterol enrichment) vs. −0.22 ± 0.22% (vehicle control) (Figure 6C,D; *p* = 0.537 in Mann–Whitney U nonparametric test).

**Figure 7:**
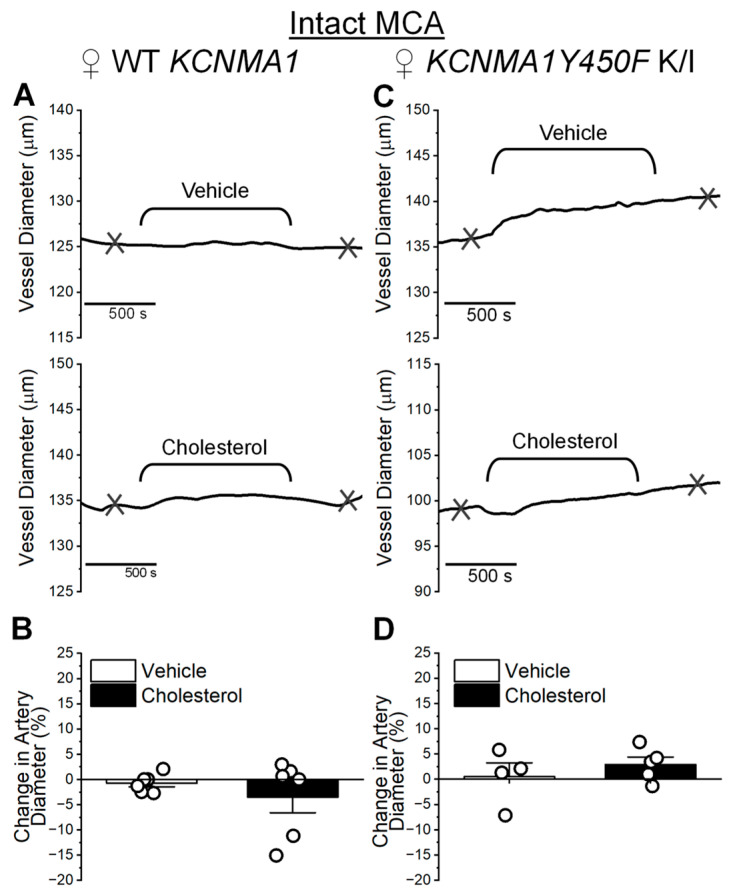
Cholesterol enrichment fails to alter diameter of middle cerebral arteries (MCAs) from female mice of WT *KCNMA1* and *KCNMA1Y450F* K/I genotypes. (**A**) Representative traces of MCA diameter over perfusion with 5 mM MβCD (vehicle, top trace) or 6.25 mM cholesterol/5 mM MβCD (cholesterol, bottom trace) solutions. MCA was dissected out of female WT *KCNMA1* mouse and pressurized at 60 mmHg. Integrity of the endothelial layer was preserved. Here, and in (**C**), crisscross marks depict time points at which diameter measurements were obtained for plotting. Measurement of base diameter was obtained prior to perfusing pressurized artery with vehicle or cholesterol-enriching solution. Measurements of the effect of cholesterol-enriching perfusion and time-matched vehicle control were obtained 10 min following washout of respective solutions from the pressurized artery chamber. (**B**) Bar graph shows average and individual artery responses to vehicle or cholesterol-enriching solutions. MCAs were obtained from female WT *KCNMA1* mice. Here and in (**D**), change in artery diameter shows change in diameter triggered by vehicle perfusion or cholesterol enrichment as percent of base diameter before perfusion with vehicle or cholesterol-enriching solution. (**C**) Representative traces of MCA diameter over perfusion with 5 mM MβCD (vehicle, top trace) or 6.25 mM cholesterol/5 mM MβCD (cholesterol, bottom trace) solutions. MCA was dissected out of female *KCNMA1Y450F* K/I mouse and pressurized at 60 mmHg. (**D**) Bar graph shows average and individual artery responses to vehicle or cholesterol-enriching solutions. MCAs were obtained from female *KCNMA1Y450F* K/I mice.

The same myogenic tone experiments were performed on female MCAs. As found with cholesterol enrichment of de-endothelialized MCAs from WT *KCNMA1* and *KCNMA1Y450F* K/I female mice (Figure 5), the diameter of female MCAs with an intact endothelium remained unchanged by cholesterol enrichment (Figure 7). Diameter changes in MCAs from WT *KCNMA1* females averaged −3.50 ± 3.11% (cholesterol enrichment) and −0.73 ± 0.73% (vehicle control) (*p* = 0.818 in Mann–Whitney U nonparametric test) (Figure 7A,B). Similarly, *KCNMA1Y450F* K/I did not exhibit any sensitivity to cholesterol, with averaged changes in diameter of 2.94 ± 1.48% (cholesterol enrichment) and −0.67 ± 6.48% (vehicle control) compared to the diameter before cholesterol enrichment or vehicle application, respectively (*p* = 0.667 in Mann–Whitney U nonparametric test) (Figure 7C,D).

## 3. Discussion

The high intake of cholesterol within Western diets is associated with an elevated prevalence of cardiovascular, including cerebrovascular, diseases. An estimated 38.1% of adults in the US have elevated plasma cholesterol [29]. Abnormal cholesterol levels contribute to the pathophysiology of stroke [30,31] and cognitive deficits including some forms of dementia [32]. Of note, cerebral arteries are resistant to the formation of atherosclerotic plaques [33], but their membrane cholesterol concentration increases during a high-cholesterol diet, eventually leading to pathology [22,34,35,36,37]. Thus, understanding the molecular mechanisms by which elevated cholesterol exerts its physiological and pathological effects may have a great impact on public health. Framed within this relevance, and based on the critical role of SM BK channels in controlling the SM tone and diameter of cerebral arteries (reviewed in [38]), in this work, we aimed at determining the role of a specific residue (Y450) in the BK channel-forming α subunit in controlling both basal function and the cholesterol responses of BK channels when probed in their native SM cells, as well as the importance of that single amino acid for cholesterol action on the cerebral artery diameter. Our focus on slo1 Y450 is based on previous data from binary phosphoglyceride bilayers showing that cholesterol at concentrations that match those in native plasma membranes (33 mol%) decreases both the channel-open probability (Po) and unitary current amplitude of homotetrameric slo1 channels [23,24,39], with this reduction in Po being significantly ablated by the Y450F substitution [24]. Considering that the BK channel consists of several subunit types with differential half-life of membrane presence [40], and that one of the BK channel subunits has been shown to mobilize towards the membrane upon cholesterol enrichment [25], it is critical to preserve intracellular protein trafficking machinery in studies of native BK channels. Thus, this study allows for a tighter comparison of cholesterol’s overall effect on BK channel-mediated currents in vascular smooth cells and cholesterol’s effect on endothelium-independent modifications of vascular diameter. For this reason, we chose a cell-attached configuration for our current electrophysiology work.

Present electrophysiological data first show that cholesterol enrichment of native MCA SM BK channels from WT male mice leads to a mild yet statistically significant reduction in unitary current amplitude (Figure 2C). This cholesterol action is consistent with previous data from slo1 channels cloned from MCA SM cells and reconstituted into binary phosphoglyceride bilayers in the presence of 33 mol% cholesterol [39]. The similarity in outcomes of the two studies underscores that a cholesterol-induced reduction in unitary current, previously reported in a highly simplified and nonphysiological system (a binary phospholipid bilayer), is sustained in the complex proteolipid environment of native SM membranes, which includes BK regulatory channel subunits and other proteins that regulate channel function, and in the presence of cytosolic signaling, which is conserved in our current cell-attached recordings. The mechanisms that contribute to a cholesterol-induced decrease in BK channel unitary conductance have been discussed in detail elsewhere [41].

In addition, cholesterol enrichment of MCA SM cells from a WT mouse leads to a mild increase (trend) in BK current density (Po/pipette tip resistance) and a trend of decrease in unitary current amplitude (Figure 2A–C). Lack of a robust increase in current density differs from our previous work reporting an increase in cerebrovascular BK current upon cholesterol enrichment of MCA myocytes from WT *KCNMA1* mice, which has been attributed to cholesterol-induced shuttling of BK regulatory β1 subunits from intracellular compartments to the plasmalemma [25]. The quantitative difference in outcome between previous and current results may stem from differences in data acquisition: Bukiya et al. [25] recorded BK currents from channels whose cytosolic calcium sensors were exposed to free calcium buffered to 30 μM (bath solution; inside-out configuration). In contrast, our current data were obtained in the cell-attached configuration. Even in contracting myocytes, intracellular calcium levels hardly reach 30 μM and cannot be tightly titrated [42]. Since the activatory action of cholesterol is related to an increased availability of β_1_ subunits in the plasmalemma [25], and the amplificatory effect of these subunits on channel activity increases with calcium (particularly evident at ≥1 μM [43]), the low in our recordings likely contributes to the reduced potentiation of SM BK currents by cholesterol.

It could be argued that the lack of robust sensitivity of WT *KCNMA1* mouse cerebral artery myocyte BK channels to cholesterol enrichment might be due to under-enrichment of myocyte detergent-resistant membrane domains with cholesterol (Figure 1B). Indeed, BK channels were reported to reside in the detergent-resistant fraction of smooth muscle cellular membranes [44]. However, Lam et al. showed that treatment of colonic epithelial cells with MβCD caused the movement of BK channels from detergent-resistant to detergent-sensitive membrane fractions [45]. While we can only assume that myocyte treatment with the MβCD/cholesterol complex would promote BK channel migration in the opposite direction (from detergent-sensitive to detergent-resistant domains), such migration would increase BK channel clustering in detergent-resistant membrane areas. The functional implications of BK channel lateral migration within the plasma membrane upon differential distribution of cholesterol between detergent-sensitive and detergent-resistant domains in mouse cerebral arteries, however, remains to be established.

Current electrophysiological data also show that the slo1 channel Y450F substitution reduces the BK channel unitary current amplitude (Figure 2F), likely a consequence of Y450 being located next to the second Regulator of Conductance for the potassium (RCK) domain of the adjacent α subunit, in proximity to the inner leaflet and the calcium-sensing site. Remarkably, cholesterol enrichment, rather than synergizing with the negative effect of Y450F on unitary conductance, introduces antagonism, which actually increases the unitary current amplitude of the mutant when compared to the values in the absence of cholesterol (Figure 2F). The reason for this antagonism remains to be determined.

In addition to its action on unitary current amplitude, cholesterol has been consistently reported to reduce the steady-state activity (Po) of slo1 channel homotetramers reconstituted into planar lipid bilayers [23,24,39,46]. Moreover, the slo1 Y450F substitution drastically reduces this cholesterol action on slo1 channels cloned from rat MCA SM in this simplified system [24]. BK channels in their native tissues, however, are multimeric complexes; in cerebrovascular SM cells, BK channels include regulatory β_1_ and γ subunits [47,48]. Considering (a) the central role of slo1 Y450 in participating in the inhibitory effect of cholesterol on slo1 Po and (b) cholesterol-driven mobilization of activatory β_1_ subunits to the plasma membrane (see above) with eventual increased BK channel activity, we hypothesized that, if the effect of the Y450F mutation on cholesterol action on slo1 channels is kept in native BK counterparts and cholesterol still increases β_1_-mediated activation of the BK current, MCA SM BK channels from a *KCNMA1Y450F* mouse would be potentiated by cholesterol more than SM BK channels from WT mice. Surprisingly, BK channels from male *KCNMA1Y450F* K/I myocytes are inhibited by cholesterol (Figure 2D–F). A possible explanation for this result may stem from the hypothetical involvement of slo1 channel Y450 in sensing the presence of activatory β_1_ subunits. Conceivably, the Y450F substitution prevents β_1_ subunits from interacting with channel-forming α subunits, subsequently preventing an increase in channel activity and thus current density. Indeed, due to the proximity of slo1 Y450 to the inner membrane leaflet and to the interface of adjacent α subunits, this residue may participate in the interaction with the β_1_ subunit. The latter contains two transmembrane helices and is positioned between the transmembrane regions of two conjoined α subunits [49]. Thus, slo1 Y450 may serve as an anchoring point for amino acid residues in the cytosolic region of the β_1_ subunit, or a point of contact for an unidentified third party (chaperone protein, membrane signaling messenger, etc.), which enables the functional interaction between BK α and β_1_ subunits. The hypothesis that Y450 may be enabling the interaction(s) between BK channel-forming subunits and regulatory β_1_ subunits merits investigation. Studies to follow-up on this idea may be particularly relevant considering that β_1_ subunit-containing BK channels control cerebral blood flow [6] and, therefore, that Y450F mutation could be associated with an increased risk of cerebrovascular diseases and/or could serve as a therapeutic target.

Lastly, our electrophysiological data show that the changes in basal function and cholesterol responses introduced by the *KCNMA1Y450F* point mutation are evident in MCA SM cells from a male and yet not a female mouse. As the expression of BK channel subunits and their responses to steroids other than cholesterol, including female sex hormones, exogenous estrogens, and progesterone have demonstrated sexual dimorphism in rats and mice [50,51,52,53], sexual dimorphism of the effect of cholesterol on BK currents is somewhat expected. Our data show that MCA BK current density from males responds more to cholesterol enrichment than that of females, whereas the unitary current amplitude response to cholesterol is identical for the sexes (Figure 2C,F and Figure 3C,F). This dichotomy is consistent with the fact that modulation of channel activity, including by sex hormones, is prevalent over regulation of ion conductance. Sexual dimorphism in the effect of the slo1 channel Y450F substitution on BK current sensitivity to cholesterol enrichment suggests a potential role for this residue in tuning BK channel pharmacology to sex-specific signaling. It is noteworthy that similar sex-specific consequences (mediated by individual residues) have been described in differentially spliced slo1 channel isoforms of the parasitic nematode filariae: the anthelmintic compound emodepside acts on the female slo1 isoform with higher potency than on the male isoform due to Asp/Glu567 and Thr/Ser568 (males/females, respectively), residues found in the drug-binding pocket [54]. While further studies are necessary for a full understanding of the mechanisms which contribute to sexual dimorphism in BK current response to cholesterol enrichment, it can be expected that sex hormones play a role. Previous studies have shown sexual dimorphism of vascular SM BK channels, including responses to treatment with other steroid molecules [50,53], yet this could be attributed to a wide variety of factors, including differences in protein and/or transcript expression of α or β subunits, differences in expression of other proteins, and/or differences in intracellular calcium levels.

Considering that (a) BK channels in the vascular SM itself have a major role in tissue and organ physiology (in particular, affecting SM tone and vascular diameter [38,47]) and (b) the vast majority of the cell count in cerebral arteries is provided by SM cells [3], we initially evaluated in de-endothelialized MCA segments the consequences at the organ level of BK channel modulation by cholesterol enrichment and/or *KCNMA1Y450F* mutation. Ex vivo-pressurized cerebral arteries allow for evaluation of the organ function at the physiological pressure and solution composition, and this experimental method has been widely used for studies with clinical implications [55,56,57]. In de-endothelialized MCAs, we failed to detect diameter changes in response to cholesterol enrichment of MCAs from male WT *KCNMA1* mice (Figure 4A,B). Noteworthy, we evaluated the effect of a 15 min cholesterol enrichment on artery diameter by quantifying artery diameter 10 min into washout of the cholesterol/MβCD complex. Removal of MβCD prior to evaluating cholesterol’s effect was essential, as cyclodextrins have been shown to block voltage-gated potassium channel currents independent of action on the cholesterol level [58]. Using rat cerebral arteries, however, we have previously shown that even 5 min incubation of arteries in the cholesterol/MβCD complex is enough to render a statistically significant increase in cholesterol level [59]. In our present work, 15 min incubation of mouse MCAs resulted in a several-fold increase in tissue cholesterol level in arteries from both male and female WT *KCNMA1* mice (Figure 1). Moreover, it has been found that elevated cholesterol levels persist for up to 2 h following washout of the cholesterol/MβCD complex [59]. In this scenario, cholesterol levels are still elevated even after washout of the cholesterol/MβCD complex from the artery perfusion chamber. The lack of change in diameter of de-endothelialized MCAs by cholesterol enrichment in WT mice can be attributed to a few non-mutually exclusive factors. One, cholesterol exerts a dual action on BK channels: it tends to increase current density (which includes both N and Po components) while decreasing unitary current amplitude (Figure 2A–C). These opposing actions could cancel each other out, rendering no net effect on total BK current and thus no major contribution of these channels to SM tone and diameter of de-endothelialized MCA segments. A similar argument can be made regarding the lack of changes in male *KCNMA1Y450F* mouse MCAs upon cholesterol enrichment (Figure 4C,D) despite the fact that BK current density is decreased and unitary current amplitude is increased by cholesterol enrichment of myocytes from these arteries (Figure 2D–F): decreased current density is compensated for by the increased unitary amplitude. In addition, several ion channels other than BK that are present in the vasculature are sensitive to manipulations in cholesterol level [60,61,62]. For instance, elevation of cholesterol level is shown to increase the L-type voltage-sensitive calcium channel current in arterial SM cells [63], and L-type channels control BK channel activity in cerebrovascular SM (reviewed in [38]). Thus, opposite modulation of ion channels that control SM tone could lead to there being no net effect on MCA diameter.

Although the functional role of BK channels in cerebral artery endothelium is debated, given the many regulatory mechanisms that the endothelium exerts on vascular diameter [64], we expected intact MCA segments to respond differently to cholesterol enrichment than their de-endothelialized counterparts; for example, the presence of endothelium could confer MCA sensitivity to cholesterol enrichment. Upon cholesterol enrichment, however, MCAs from WT *KCNMA1* or *KCNMA1Y450F* K/I mice, whether from male or female animals, did not exhibit changes in diameter (Figure 6). This underscores that if cholesterol enrichment of MCAs modifies endothelial function, such modification does not translate into SM tone and vessel diameter. While this is the conclusion we can draw directly from the dataset in this study, we recognize that the wide range of data may induce a type 2 statistical error, which is a limitation of this study.

In conclusion, the present study demonstrates that the slo1 Y450F substitution modifies in a sexually dimorphic manner both the basal function and cholesterol response of MCA SM BK channels when studied in their native cells, yet such modifications do not translate into an artery diameter response to cholesterol.

## 4. Materials and Methods

### 4.1. Animals

For all animal experiments, 8- to 12-week-old *KCNMA1* WT and *KCNMA1Y450F* K/I mice on a C57BL/6J background were bred in-house. WT and K/I animals came from the same progenitors, having been born upon backcrossing heterozygous mice. Female mice were used in non-estrus phases to avoid a dip in estrogen levels. On the day of the experiment, mice were deeply anesthetized with isoflurane (open-drop method; Covetrus, Dublin, OH, USA) via inhalation and decapitated with sharp scissors. Middle cerebral arteries were isolated on ice from the mouse brains and cleaned of surrounding tissue.

The care of animals and experimental protocols were reviewed and approved by the Institutional Animal Care and Use Committee of the University of Tennessee Health Science Center (protocol number 23-0420, approved on 16 February 2023). The university is an institution accredited by the Association for Assessment and Accreditation of Laboratory Animal Care.

### 4.2. Cholesterol Quantification

Middle cerebral arteries were dissected from the brains of 8–12-week-old male C57BL/6J mice. Arteries were kept in cold physiological saline solution (PSS) except for a 15 min incubation in cholesterol-enriching (6.25 mM cholesterol in 5 mM MβCD [59]) or vehicle control (5 mM MβCD) solution. PSS has the following composition (mM): 119 NaCl, 4.7 KCl, 1.2 KH_2_PO_4_, 1.6 CaCl_2_, 1.2 MgSO_4_, 0.023 EDTA, 11 glucose, 24 NaHCO_3_. PSS was equilibrated at pH 7.4 with a 21/5/74 percentage mix of O_2_/CO_2_/N_2_ prior to use. After vehicle or cholesterol treatment, arteries were washed with PSS for 5 min. Tissue was lysed with RIPA buffer (ThermoFisher, Waltham, MA, USA, 89901) and 1% protease inhibitor (ThermoFisher, Waltham, MA, USA, 78429), then sonicated with periodic vortexing (Fisher Scientific, Pittsburgh, PA, USA, Sonic Dismembrator Model 100). For the measurement of total cholesterol amount, lysate was then diluted 1:3 in PSS, and total protein was quantified using the Pierce BCA (bicinchoninic acid) Protein Assay Kit according to the manufacturer’s protocol (ThermoFisher, Waltham, MA, USA, 23225). Next, lysate was diluted 1:3 in 1% Triton X-100 (Fisher Scientific, Pittsburgh, PA, USA, BP151) in PSS.

For measuring cholesterol amounts in detergent-sensitive versus detergent-resistant membrane fractions, cerebral arteries from the Circle of Willis (anterior, middle, posterior, and basilar) were dissected from male mouse brains. Arteries were lysed in 1% Triton X-100 in PBS with 1% protease inhibitor. Cholesterol in detergent-sensitive and detergent-resistant fractions was quantified as described by our group [22]. In short, the detergent-sensitive fraction was collected following a brief 21,000× *g* centrifugation. The cellular debris remaining after detergent-sensitive fraction collection was reconstituted into a sucrose gradient and centrifuged for 16 h at 220,500× *g* using a Beckman (Brea, CA, USA) SW55TI rotor to collect the detergent-resistant fraction.

Cholesterol measurements were performed using the Amplex Red Cholesterol Assay Kit following the manufacturer’s protocol (ThermoFisher, Waltham, MA, USA, A12216). Cholesterol concentration was normalized to grams of protein in each sample.

### 4.3. Myocyte Isolation and Voltage-Clamp Electrophysiological Recordings

Myocytes were isolated from middle cerebral arteries as described previously by our group [28,53,65]. Briefly, arteries were enzymatically isolated by two-step enzymatic digestion with 0.03% papain followed by 2% collagenase. Arteries were then manually dissociated with a set of glass pipettes featuring decreasing tip diameters. Resulting myocytes were placed on a poly-D-lysine-treated dish for 10 min to allow for cell adhesion to the bottom of the plate. Then, the plate was filled with the treatment solution (5 mM MβCD as a vehicle or 6.25 mM cholesterol in 5 mM MβCD as a cholesterol-enriching solution) for 15 min. The plate was then washed with bath solution and immediately used for patch-clamp experiments. Cells were used up to 1 h following vehicle or cholesterol treatment. During this time, elevation of the cellular cholesterol level persists despite the washout of the cholesterol enrichment treatment (Figure 1) [59]. Bath solution contained (mM): 130 KCl, 2.97 CaCl_2_, 1 MgCl_2_, 5 EGTA, 15 HEPES; pH 7.4; 0.1 μM [Ca^2+^]_free_. The high potassium bathing solution was used to zero the resting potential; thus, V_m_ = −V_pipette_. Pipette solution contained (mM) 127 NaCl, 3 KCl, 1.8 CaCl_2_, 2 MgCl_2_, and 15 HEPES; pH 7.4.

Ionic currents were obtained using the cell-attached configuration at a single-channel resolution over a range of applied voltages using an EPC8 amplifier (HEKA, Holliston, MA, USA) in the gap-free mode (1 kHz filtering, 5 kHz acquisition) for 20 s per voltage. Data were digitized using an Axon Digidata 1550B analog/digital converter (San Jose, CA, USA) and pCLAMP 11.1 software (Molecular Devices, San Jose, CA, USA). Current density was calculated from the channel-open probability (NPo) obtained using Clampfit 10.7 (Molecular Devices, San Jose, CA, USA) divided by individual pipette tip resistance.

### 4.4. Cerebral Artery Diameter Measurement Ex Vivo

Middle cerebral arteries were isolated from mouse brains [28,65] and cannulated at each end in a vessel perfusion chamber (Living Systems Instrumentation, St. Albans, VT, USA, CH-1). For de-endothelialized arteries, endothelium was removed by passing an air bubble through the vessel lumen for 90 s before the second end of the artery was cannulated [28]. Throughout the experiment, the chamber was perfused with 35–37 °C PSS using a peristaltic pump. Artery diameter was visualized using a charged-coupled device camera attached to an inverted microscope and traced using the automatic edge-detection function of IonWizard software (v6.6, IonOptix, Westwood, MA, USA) digitized at 1 Hz. Intravascular pressure was monitored using a pressure transducer (Living Systems Instrumentation, St. Albans, VT, USA, PT-F) and perfusion pressure monitor (Living Systems Instrumentation, St. Albans, VT, USA, PM-4). The artery was first pressurized to 10 mmHg for 10 min and then 60 mmHg for the remainder of vessel diameter acquisition. Drug perfusion (5 mM MβCD or 6.25 mM cholesterol/5 mM MβCD in PSS) began following myogenic tone development of the artery in response to 60 mmHg, and then it was washed out with PSS. Maximal contractility was determined using a depolarizing 60 mM KCl solution. Arterial segments that failed to constrict to KCl were not included for analysis. Depolarizing KCl solution was composed of (mM) 63.7 NaCl, 60 KCl, 1.2 KH_2_PO_4_, 1.2 MgSO_4_, 0.023 EDTA, 11 glucose, 24 NaHCO_3_, and 1.6 CaCl_2_ (pH = 7.4 by 21/5/74 percentage mix of O_2_/CO_2_/N_2_). Measurement of vehicle or cholesterol enrichment effects was calculated by dividing the vessel diameter over 10 min of either washout with vehicle or cholesterol-enriching treatment by the diameter just prior to vehicle or cholesterol-enriching treatment, respectively.

### 4.5. Chemicals

Cholesterol (C8667), methyl-β-cyclodextrin (C4555), soybean trypsin inhibitor (T9129), collagenase (C8051), calcium chloride (C3881), sodium bicarbonate (S6014), phenol red (P4633), papain (P4762), and taurine (T8961) were obtained from Sigma-Aldrich (St. Louis, MO, USA). Magnesium sulfate (MX0070-1) and paxilline (P2928) were purchased from EMD Millipore Sigma (Burlington, MA, USA). BSA (001-000-162) was acquired from Jackson ImmunoResearch (West Grove, PA, USA). Ethylenediamine tetra-acetic acid (EDTA) (0.5 M; E177) was purchased from VWR (Solon, OH, USA). Sodium chloride (S271), potassium chloride (P217), potassium phosphate (P285), and magnesium chloride (M33) were purchased from Fisher Scientific (Pittsburgh, PA, USA). The clinical blood gas mixture (5.0% carbon dioxide, 21% oxygen, and 74% nitrogen; UN1956) was purchased from Nexair (Memphis, TN, USA).

### 4.6. Statistical Analysis

All data were displayed and plotted using Origin software (v2022b, OriginLab Co., Northampton, MA, USA). Outliers were removed according to Grubb’s test for outliers with a co-efficient of 1 using the built-in function in Origin software (v2022b). Nonparametric Mann–Whitney nonparametric and two-way ANOVA tests were performed using SPSS (v28, IBM, Armonk, NY, USA). All testing assumed two-tailed p-values with significance set at *p* < 0.05. Data are presented as the mean ± SEM.

## Data Availability

The original contributions presented in this study are included in the article/Supplementary Materials. Further inquiries can be directed to the corresponding author.

## Supplementary Materials

The following supporting information can be downloaded at https://www.mdpi.com/article/10.3390/ijms26083814/s1.

## Abbreviations

The following abbreviations are used in this manuscript:

BK	Voltage- and calcium-gated potassium channel of large conductance (“Big K^+^”)
SM	Smooth muscle
WT	Wild type
K/I	Knock-in
MCA	Middle cerebral artery
Po	Channel-open probability
MβCD	Methyl-β-cyclodextrin
RCK	Regulator for conductance of potassium
