# Supplementary material for: The Slo1 Y450F Substitution Modifies Basal Function and Cholesterol Response of Middle Cerebral Artery Smooth Muscle BK Channels in a Sexually Dimorphic Manner
Source: Int J Mol Sci. 2025 Apr 17;26(8):3814. doi: 10.3390/ijms26083814 (PMC12028221; doi:10.3390/ijms26083814)
Supplement: Supplementary file 1 [file ijms-26-03814-s001.zip › ijms-3559536-supplementary.pdf]

Supplementary Materials

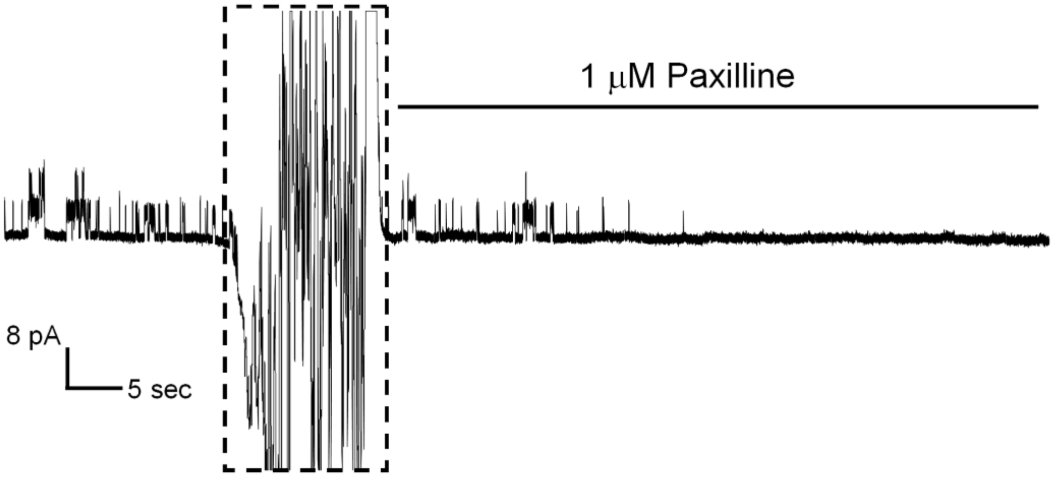

Figure S1. Paxilline blocks currents observed in cell-attach patches of mouse MCA myocytes. Original current recording obtained in cell-attached patch configuration at +60 mV from male mouse MCA myocyte. Activity of observed channels is blocked following administration of 1 μM paxilline, a selective BK channel blocker [1]. Dashed rectangle frames the noise introduced to continuous recording upon manual addition of paxilline into the patch-clamp plate using laboratory pipette.

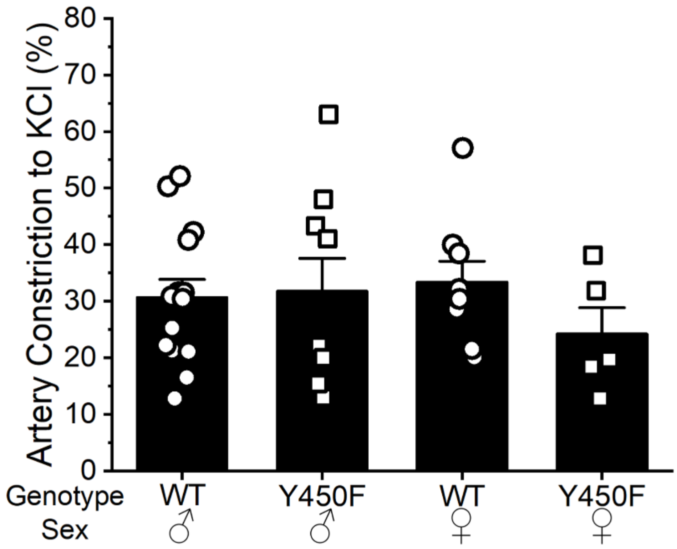

Figure S2. Summary plot showing constriction of middle cerebral arteries in response to 60 mM KCl. All arteries were perfused with 60 mM KCl following probing with vehicle or cholesterol-enriching solution. Artery constriction by KCl reflects percent decrease in artery diameter during KCl application when compared to artery diameter immediately prior to KCl perfusion. Only arteries which constricted 10% or more during perfusion with KCl were included in data analysis. Two-way ANOVA statistical testing failed to detect significant effect of sex or genotype on artery constriction by KCl.

Reference

1. Zhou, Y.; Lingle, C.J. Paxilline inhibits BK channels by an almost exclusively closed-channel block mechanism. *J. Gen. Physiol.* **2014**, *144*, 415–440. https://doi.org/10.1085/jgp.201411259.
